# Metabolic syndrome among type 2 Diabetes Mellitus patients in Ethiopia: a systematic review and meta-analysis

**DOI:** 10.3389/fcdhc.2024.1437288

**Published:** 2024-11-28

**Authors:** Tesfaye Getachew Charkos, Hunde Lemi, Godana Arero, Menberu Getnet

**Affiliations:** ^1^ School of Public Health, Adama Hospital Medical College, Adama, Ethiopia; ^2^ Department of Internal Medicine, Adama Hospital Medical College, Adama, Ethiopia

**Keywords:** diabetes mellitus, metabolic syndrome, prevalence, meta-analysis, ethiopia

## Abstract

**Introduction:**

The prevalence of metabolic syndrome among type 2 diabetes mellitus patients was inconsistent in Ethiopia. Therefore, we aimed to pool the prevalence of metabolic syndrome among type 2 diabetes mellitus patients using a systematic review and meta-analysis.

**Methods:**

PubMed, EMBASE, and Cochrane Library databases were systematically searched for relevant articles from January 2023 to January 2024. In addition, a manual search was conducted using published articles’ reference lists. The random-effects model was used to pool prevalence from individual studies. All analysis was performed using R software.

**Results:**

A total of nine articles met the inclusion criteria and were included in the analysis. The participants’ average age was 59.8 ± 3.84 years old. The pooled prevalence of MetS in T2DM patients was 53% (95% CI: 47–58). A significant heterogeneity was found across the included studies (P < 0.001, I2 = 92%). Based on diagnostic criteria, the prevalence of MetS in T2DM patients was 49% (95% CI: 43–56), 57% (95% CI: 47–67), 57% (95% CI: 43–77), and 44% (95% CI: 20-58) based on IDF, NCEP-ATP II, 2009 harmonized, and WHO criteria. By gender, the prevalence of MetS in T2DM patients was 48% (95% CI: 28–68) for females and 32% (95% CI: 17–49) for males.

**Conclusion:**

This study found that over half of type 2 diabetes mellitus patients in Ethiopia are affected by metabolic syndrome, with a higher prevalence observed in females compared to males. The NCEP-ATP II and 2009 harmonized criteria consistently yielded similar prevalence rates of metabolic syndrome. These findings highlight the importance of educating T2DM patients on preventing and managing cardiovascular disease and its related complications.

## Introduction

Metabolic syndrome (MetS) was defined as the presence of three or more of the following five risk factors: elevated fasting plasma glucose, high blood pressure, abdominal obesity, low HDL cholesterol, and/or elevated plasma triglycerides (1). It is a condition that increases the risk of coronary heart disease, diabetes, stroke, and other serious health issues, also known as insulin resistance syndrome (2–4). Moreover, it is primarily characterized by insulin resistance, obesity, dyslipidemia, and hypertension (5). MetS and type 2 diabetes mellitus (T2DM) are closely related. Their main consequences in the general population increase the risks of cardiovascular disease (CVD), stroke, and death due to cardiovascular complications (6). On the other hand, T2DM is a specific metabolic disorder characterized by chronic hyperglycemia due to insulin resistance or insufficient insulin production (7). While individuals with MetS are at a higher risk of developing T2DM (4, 7).

Noncommunicable diseases, such as metabolic syndrome, have become a global epidemic and a serious public health concern as a result of modernization and industrialization (8). Recent evidence suggests that the elevation of metabolic syndrome has raised various thoughts and considerations within the scientific community. Patients with type 2 diabetes mellitus have a high risk for metabolic syndrome due to a combination of multifactorial risk factors (T2DM). Research findings indicate that metabolic syndrome (MetS) among individuals with diabetes is influenced by various risk factors, such as advanced age (9–13), urban residence (14, 15), cigarette smokers (14), lack of adequate meal plan (11, 14, 15), BMI (9, 11, 13, 16, 17), palm oil users (14, 15), physical inactivity (12, 13, 15, 18), waist circumference (19, 20), systolic blood pressure (14, 19–21), diastolic blood pressure (20, 21), triglyceride (19–21), low high-density lipoprotein (20, 21), family history of diabetes (9, 13, 17), gender (9, 11–13, 17–19, 22), hyperuricemia (12), cholesterol (12), and any chronic disease (13).

Globally, an estimated 25% of the general population and 70–80% of patients with type 2 diabetes (T2DM) have metabolic syndrome (MetS) (23). In Sub-Saharan Africa, the prevalence of MetS among T2DM patients is 59.62% (24), while in Ethiopia, it reaches as high as 70.1% (12, 13, 25). Several studies focusing on gender revealed that MetS was quite frequent, with the prevalence ranging from 7.9% to 43% in men and 7% to 56% in women (26). Similar to this, previous studies conducted in Ethiopia revealed that, depending on various diagnostic criteria, the prevalence of MetS in men and women ranged from 16–42% in men and 25–61% in women (13, 17, 18). Moreover, the prevalence reported in the most recent study remains inconsistent (12–15, 17, 18, 22, 25). One of the primary reasons for discrepancies in results between different studies is the absence of a common set of metabolic syndrome diagnostic guidelines. In addition, a recent meta-analysis of eight cross-sectional studies reported a prevalence of 64.49% and 52.38% using the NCEP/ATP III and IDF criteria, respectively (27). However, the recent meta-analysis utilized only two criteria and included eight articles, while the current meta-analysis incorporates four commonly used criteria for assessing metabolic syndrome. This review provides comprehensive, updated insights relevant to the Ethiopian population and scientific community, offering evidence-based guidance to inform health policymakers and develop targeted intervention strategies for the emerging health challenge of metabolic syndrome in type two diabetic mellitus patients.

## Materials and methods

The current study was conducted based on the guidelines of Preferred Reporting Items for Systematic Reviews and Meta-Analyses (PRISMA) (28). This study was conducted to pool the prevalence of metabolic syndrome among type 2 diabetes patients in Ethiopia.

### Study selection

Articles were searched in the PubMed, EMBASE, and Cochrane Library databases from January 2023 to January 2024. The following search terms were used to identify relevant articles: “metabolic syndrome” OR “MS” OR “MetS” OR “insulin resistance syndrome” OR “MtS” AND “Type 2 diabetes mellitus” OR “T2DM” AND “Ethiopia.” Additionally, a manual search was conducted on the reference lists.

### Study inclusion criteria

Studies were included in this meta-analysis based on the following criteria: (1) written in English; (2) original human studies; (3) study subjects were T2DM patients; (4) studies conducted in Ethiopia; (5) studies provided the prevalence of MetS.

### The measurement used for MetS

The International Diabetes Federation (IDF) defines metabolic syndrome (MetS) as having a waist circumference (WC) > 80 cm for women and > 94 cm for men, plus two or more of the following: blood pressure (BP) ≥ 130/85 mmHg or on treatment, triglycerides (TG) ≥ 150 mg/dl or on treatment, and low HDL cholesterol (3). The advantage of using the IDF criteria lies in its incorporation of ethnicity-specific waist circumference thresholds, which enhances sensitivity to regional obesity variations. By emphasizing central obesity as a core criterion, the IDF criteria focus on a key factor closely linked to metabolic risks. However, a shortcoming of the IDF criterion is its mandatory central obesity requirement, which may exclude individuals with other MetS components.

The National Cholesterol Education Program’s Third Adult Treatment Panel defines MetS as having at least three of five risk factors: WC > 102 cm for men and > 88 cm for women, BP > 130/85 mmHg or on treatment, fasting blood glucose (FBG) ≥ 100 mg/dl or on treatment, TG ≥ 150 mg/dl or on treatment, and HDL < 40 mg/dl for men or < 50 mg/dl for women, or on treatment (3). The NCEP ATP III criteria are widely used, featuring clear cut-off points for each component and a simplified diagnostic method. However, the criteria are less sensitive to ethnic differences and rely on universal waist circumference thresholds, which may not accurately reflect obesity across diverse populations.

The 2009 harmonized criteria define metabolic syndrome as a cluster of three or more of the following five interrelated risk factors: triglycerides (TG) ≥ 150 mg/dl, HDL cholesterol < 40 mg/dl for men and < 50 mg/dl for women, blood pressure (BP) > 130/85 mmHg, fasting blood glucose (FBG) ≥ 100 mg/dl, and waist circumference (WC) > 94 cm for men and > 80 cm for women (29). The 2009 harmonized criteria for measuring Metabolic Syndrome enhance standardization and inclusivity, their complexity and potential for overdiagnosis can pose challenges in clinical application.

The World Health Organization (WHO) defines metabolic syndrome as impaired fasting glucose (IFG) > 100 mg/dl or impaired glucose tolerance (IGT), plus two of the following: abdominal obesity (waist-to-hip ratio > 0.9 in men or > 0.85 in women, or body mass index (BMI) > 30 kg/m²), triglycerides ≥ 150 mg/dl, HDL cholesterol < 40 mg/dl in men and < 50 mg/dl in women, blood pressure (BP) ≥ 140/90 mmHg, or microalbuminuria (urinary albumin secretion rate ≥ 20 μg/min or albumin-to-creatinine ratio ≥ 30 mg/g) (30). The WHO criteria provide a comprehensive approach to Metabolic Syndrome by requiring insulin resistance as a key component and assessing both clinical and biochemical markers for a holistic view of metabolic health. However, their reliance on complex testing for insulin resistance limits practicality for routine clinical screening, making them better suited for research or specialized settings.

### Data extraction

TGC and HL independently extracted all relevant articles and identified eligible studies. Any disagreements during data extraction were resolved through discussion. The following information was extracted from each included study: first author, publication year, proportion of females, sample size, the average age of the participants in the study, components of MetS (abdominal obesity, elevated blood pressure, high triglycerides, low HDL cholesterol levels, and elevated fasting blood glucose), the prevalence of MetS, and the MetS criteria used in the study.

### Quality assessment

In this study, the Newcastle-Ottawa Scale (NOS) was used to evaluate the quality of individual studies (31). It includes a set of items and assigns a maximum of nine stars to the following parameters: selection, comparability, exposure, and outcome. The quality of the study was classified based on the stars earned: five stars or less were considered low quality, six to seven stars were considered moderate, and eight stars or more were considered high quality.

### Statistical analysis

Descriptive statistics, including the mean and standard deviation, were used. A DerSimonian-Laird random-effects meta-analysis was conducted (32) because we detected significant heterogeneity between the studies. Since prevalence values are always between 0 and 1, the prevalence of MetS in each study was transformed using the Freeman-Tukey double arcsine method, and the results were then converted back to prevalence estimates for interpretation. This transformation was applied to minimize the influence of small sample studies (33). The double arcsine transformation addresses issues such as confidence limits falling outside the 0-1 range and variance instability. The analysis was performed on the transformed proportions, using the inverse of the variance of the transformed proportion as the study weight (33).

Furthermore, subgroup analyses based on gender and diagnostic criteria were conducted. Heterogeneity across studies was assessed using Cochran’s Q-statistic test, and inconsistency was quantified using the I² statistic (34, 35). Funnel plots for visual inspection and Egger’s test were performed to detect any potential publication bias (36). A forest plot was used to present the estimated pooled prevalence of MetS and its subcomponents. All analyses were conducted using the R program (version 4.4.1; R Foundation for Statistical Computing, Vienna, Austria).

## Results

### Study characteristics

**Figure 1:**
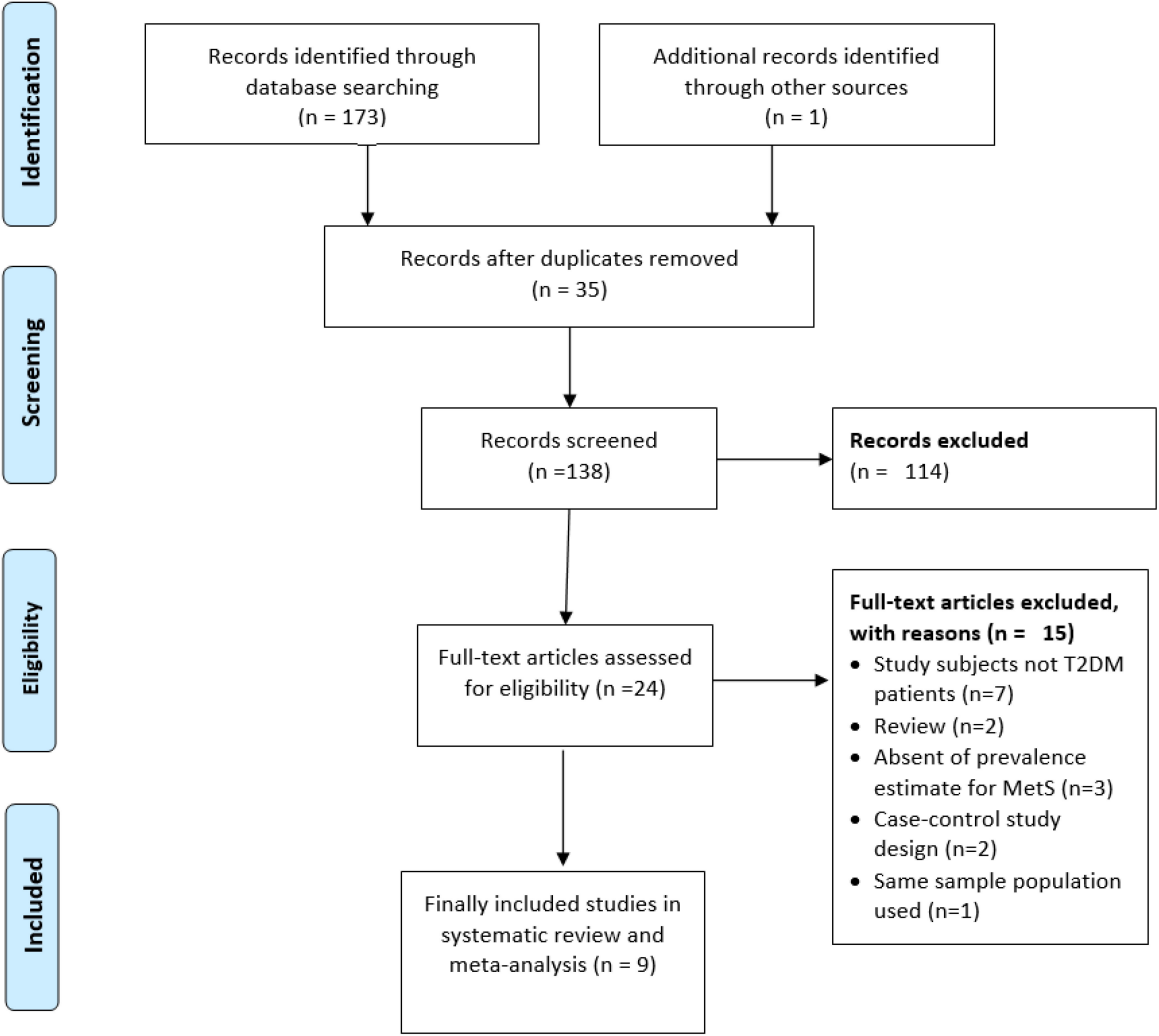
Flow chart for study inclusion and exclusions.

A flow chart summarizing the process of study selection is shown in **Figure 1**. Systematically searching for relevant studies, a total of 174 articles were identified from the electronic database search. Out of this, 35 articles were excluded due to duplicates and unrelated titles. After screening articles, 114 articles were excluded based on the title and abstract because of their irrelevance to our study aim. Twenty-four full-text articles were assessed for eligibility. Seven were excluded because the study subjects were not T2DM patients, two were review articles, three did not report the prevalence, two were excluded due to a case-control study design, and two studies used the same population data, the more recent study (12) included in this analysis and the older one (37) excluded. Finally, 9 articles with a total of 2,588 subjects were included in the final analysis (11, 13–15, 17, 18, 25) (**Table 1**). The participants’ average age was 51.88 years (SD: 3.84 years).

**Table 1:** Descriptive characteristics of all included studies.

Authors, year	Sample size		Percent of women (%)	Mean age	MetS criteria	Prevalence of components of MetS	Prevalence MetS (%)	Quality score
Gebremeskel, 2019 (13)	419		50.4	56.39 ± 10.18	IDF	Abdominal obesity (59.7%), Raised triglyceride (45.1%), Raised Hypertension (41.3%), Low HDL-c (34.4%)	51.1	7
Charkos, 2023 (14)	237		45.2	55 ± 10	IDF		53.2	7
					NCEP-ATP III	Raised blood pressure (35.9%)	41.3	
					2009 harmonized		42.8	
Gemeda, 2022 (15)	394		30.8	46.5	NCEP-ATP III		68.3	6
Tadewos, 2017 (17)	270		38.5	48.8 ± 11.9	NCEP-ATP III	Raised triglycerides (68.1%), Low HDL-c 127(47.0%), Abdominal obesity (40.7%), Raised blood pressure (28.1%)	45.9	6
Biadgo, 2018 (25)	159		59.7	49.8 ± 8.7	IDF	Central obesity (61.0%) Raised triglyceride (62.3%), Low HDL-c (32.7%), Raised blood pressure (55.4%)	53.5	7
					NCEP-ATP III	Central obesity (43.4%), Raised triglyceride (56.6%), Low HDL-c (32.7%), Raised blood pressure (55.3%)	66.7	
Shita, 2023 (18)	204		45.1	51.75 ± 11.66	IDF	Central obesity (35.3%), Raised triglyceride (25.5%), Low HDL-c (25.5%), Raised blood pressure (74.5%)	31.4	7
					NCEP-ATP III	Central obesity (19.6%)	41.2	
Zerga, 2020 (11)	330		48.5		IDF	Raised blood pressure (66.4%) Low HDL-c (48.5%)	50.3	7
					NCEP-ATP III	Raised triglyceride (51.5%), High WC (34.8%), Overweight (37.6%)	59.4	
					2009 harmonized		64.5	
Wube, 2019 (12)	319		33	49.8 ± 9.8	IDF	Abdominal obesity (18.8%)	59.9	7
					NCEP-ATP III	Raise blood pressure (25.5%)	70.5	
					2009 harmonized		65.5	
					WHO		31.2	
Birarra and Gelayee, 2018 (22)	256		55.9		IDF	Abdominal obesity (61.7%), Raised triglyceride (67.6%), Low HDL-c (66.8%)	45.3	7
					NCEP-ATP III	Raied triglyceride (68.8), Abdominal obesity (53.5%), Low HDL-c (67.2%)	70.3	
					WHO		57	

IDF, International Diabetes Federation; NCEP-ATP III, The National Cholesterol Education Program’s Third Adult Treatment Panel; WHO, World Health Organization; MetS, metabolic syndrome; mean age, mean ± standard deviation; WC, waist circumference. The prevalence of MetS in T2DM patients.

The results of methodological quality assessment tools indicate that all nine articles had a moderate or above quality (11–15, 17, 18, 22, 25). Among the included studies, the highest prevalence of MetS was 70.5% based on 2009 harmonized criteria (12) and the lowest was 31.4% based on IDF (18). The characteristics of the studies selected for this meta-analysis are presented in **Table 1**.

**Figure 2:**
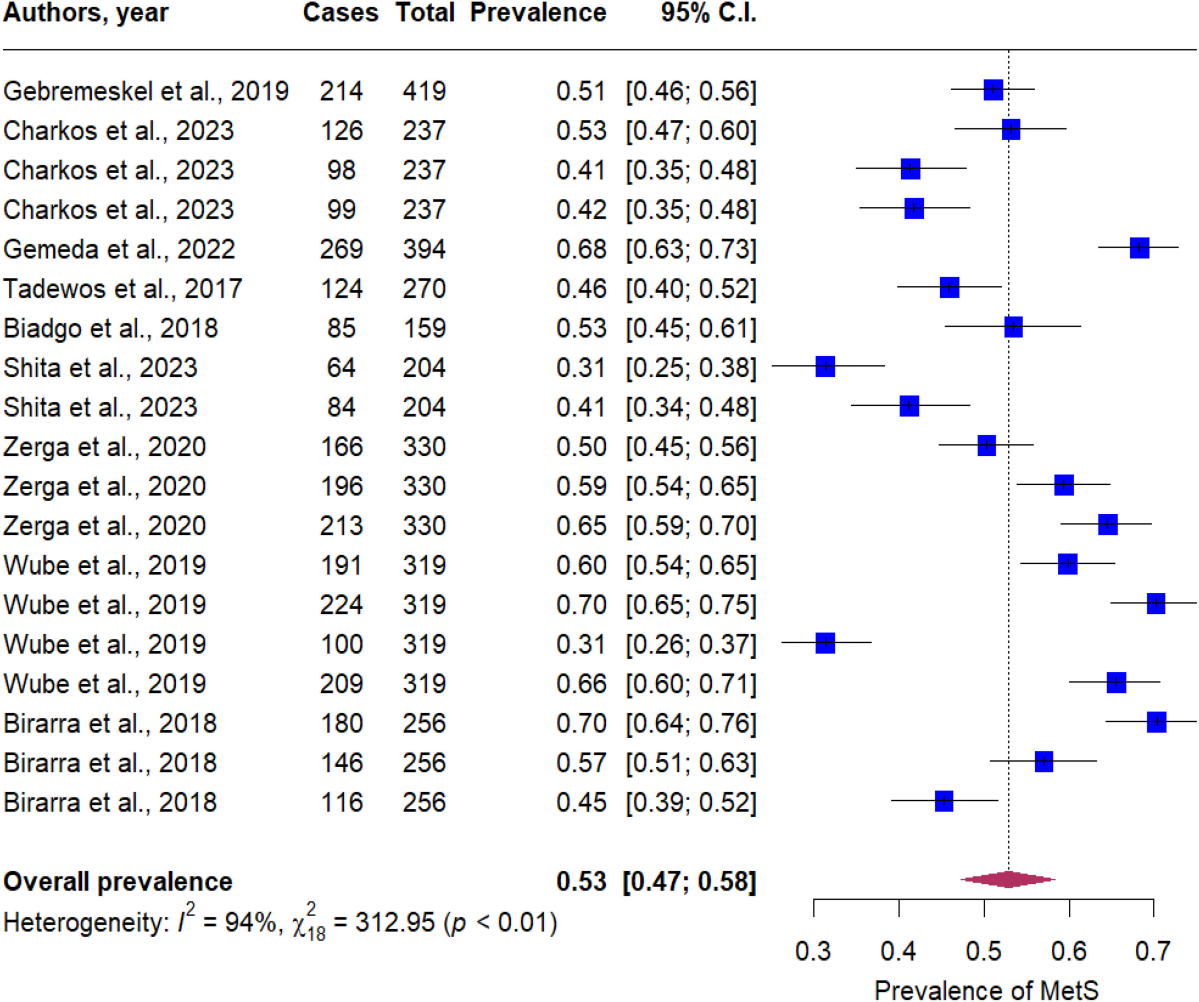
Forest plot of the prevalence of metabolic syndrome in T2DM patients in Ethiopia.

**Figure 3:**
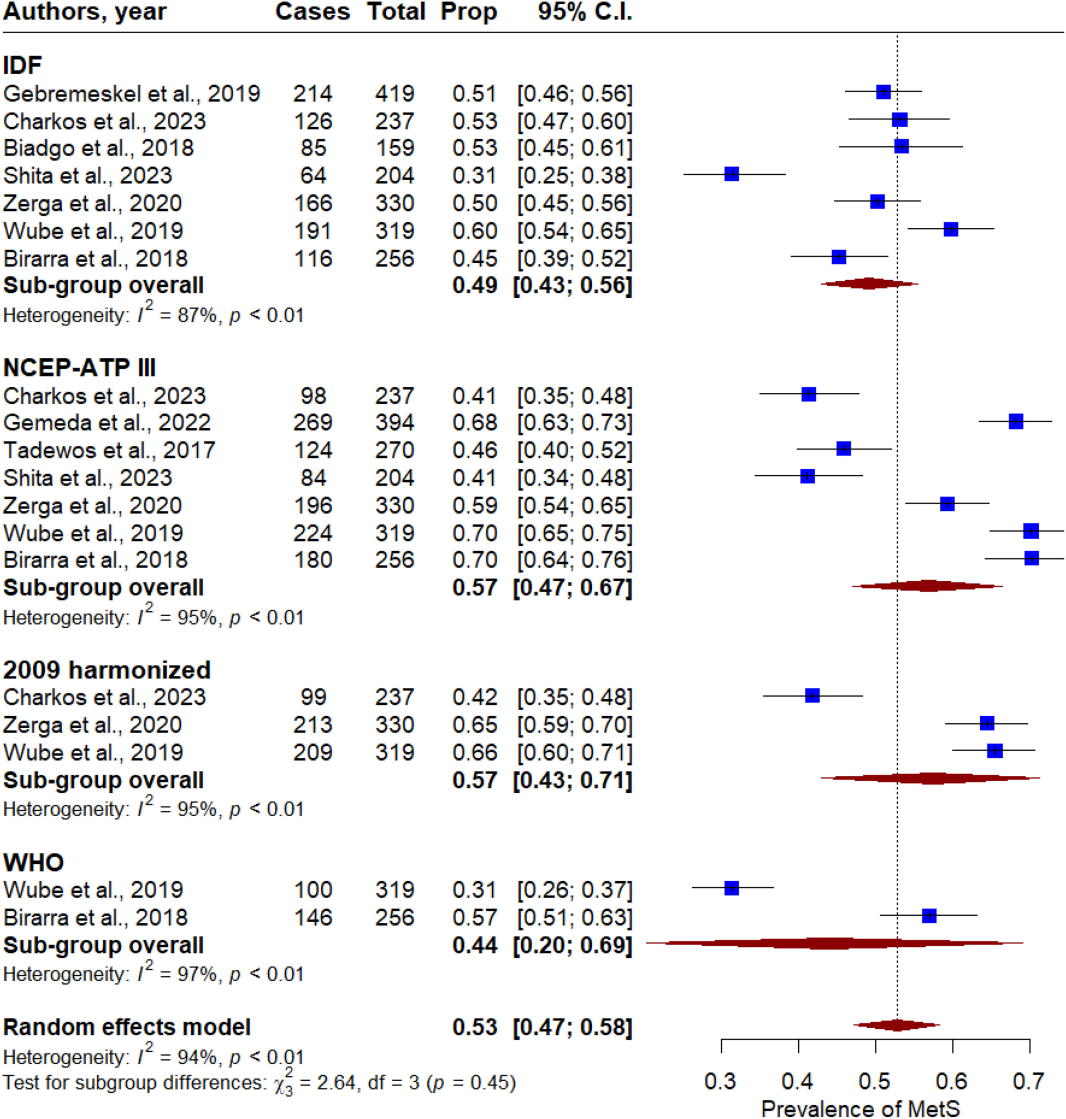
Forest plot of the prevalence of metabolic syndrome in T2DM patients in Ethiopia based on the metabolic syndrome measurement criteria.

**Figure 4:**
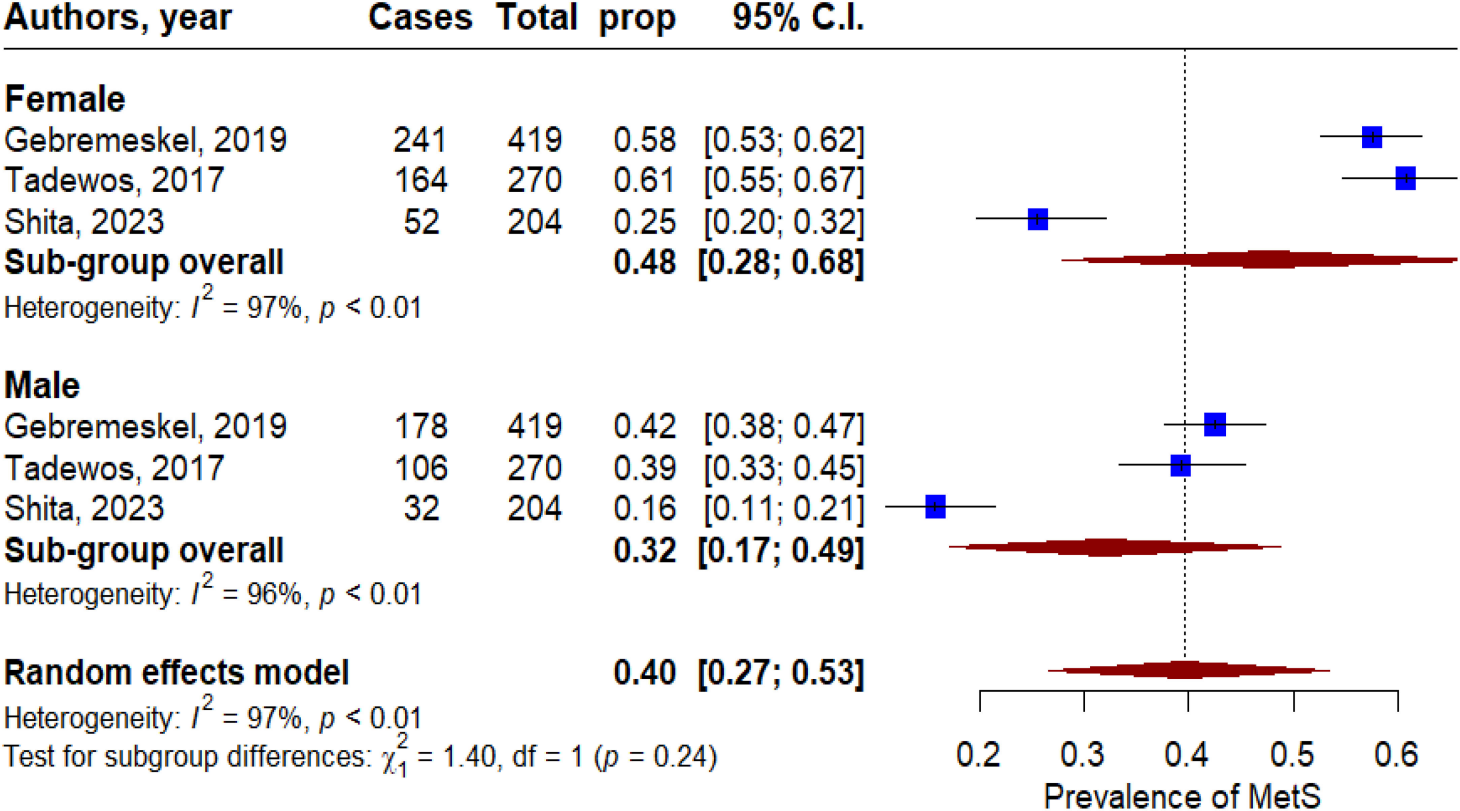
Forest plot of the prevalence of metabolic syndrome in T2DM patients based on gender.

Overall, the pooled prevalence of MetS in T2DM patients was 53% (95% CI: 47 - 58). Significant heterogeneity was observed across the included studies (P < 0.001, I^2^ = 92%, **Figure 2**). In subgroup analysis, the pooled prevalence of MetS in T2DM patients was 49% (95% CI: 43 - 56), 57% (95% CI: 47 - 67), 57% (95% CI: 43 - 71), and 44% (95% CI: 20-69) based on the IDF, NCEP-ATP II, 2009 harmonized, and WHO criteria, respectively (**Figure 3**). By gender, MetS was more prevalent in females (48%) than in males (32%) (**Figure 4**). Among the five defining components of metabolic syndrome (MetS), elevated blood pressure, increased triglyceride levels, and abnormal central obesity were the most frequently observed. These components were consistently prevalent across studies, underscoring their critical role in the diagnosis and risk assessment of MetS.

### Publication bias

**Figure 5:**
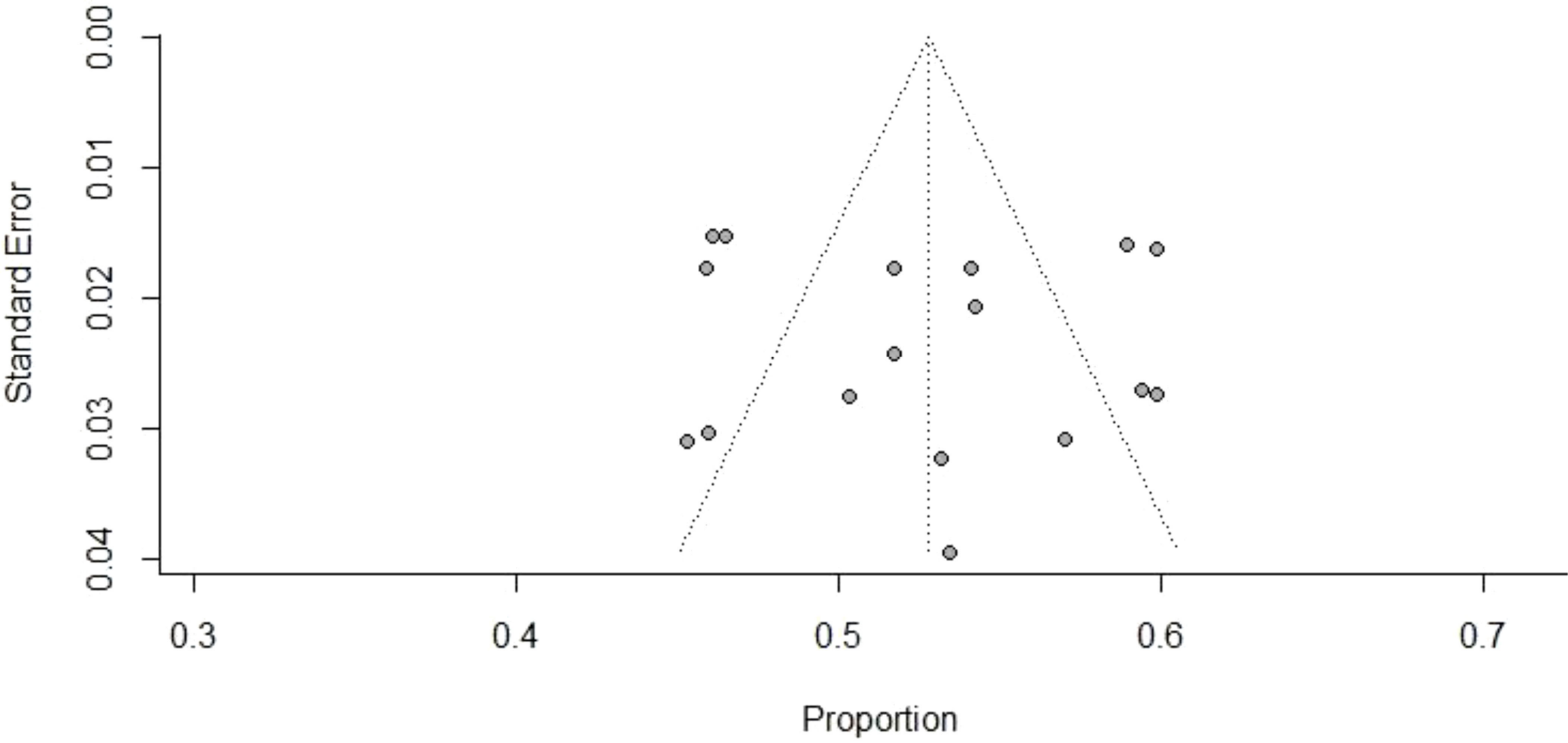
Funnel plot of publication bias, proportion versus standard error.

The funnel plot visualization demonstrates that there was no asymmetry among the included studies (**Figure 5**). Additionally, no statistically significant evidence of publication bias was found using Egger’s test (P = 0.29).

## Discussion

This systematic review and meta-analysis aimed to determine the pooled prevalence of metabolic syndrome among type 2 diabetes mellitus patients in Ethiopia. A total of nine studies, involving 2,588 men and women, were included in the final analysis. The prevalence of MetS across these studies varied significantly, ranging from 31% to 70%. The highest prevalence of MetS was 70.5% based on 2009 harmonized criteria (12) and the lowest was 31.4% based on IDF (18). Several previous studies on metabolic syndrome have suggested that the wide variation in prevalence is largely due to differences in MetS definitions or measurement criteria (38–40), a report that is supported by the current study. Additionally, factors such as participant age, gender distribution, and the sampling design used in the studies may also contribute to these observed discrepancies.

We found that the pooled prevalence of MetS among type 2 diabetes mellitus patients was 53% (95% CI: 47-58), regardless of the diagnostic criteria used for metabolic syndrome. Our findings are consistent with those of previous systematic reviews and meta-analyses conducted in sub-Saharan countries (24), and a recent study in Ethiopia reported a prevalence of 64.49% (95% CI: 62.39–66.59) using the NCEP/ATP III criteria and 52.38% (95% CI: 50.05–54.73) using the IDF (27). Similarly, the review conducted in Europe suggested that the prevalence of MetS among T2DM patients ranged between 3% and 71.7% (41). However, our findings were higher than those of previous reviews in Africa, which reported a prevalence of 32.4% (10). This discrepancy could be explained by several factors, including variations in diagnostic criteria, differences in ethnicity, and potential cultural differences, which are considered significant factors in the general population (11, 13–15, 17, 18, 25, 42).

In the subgroup analysis, the highest prevalence of metabolic syndrome was 64.8% (95% CI: 54.74, 74.86) reported using the NCEP ATP III criteria (24). Consistently, in the current study, we found that the prevalence of metabolic syndrome was 57% using both the NCEP-ATP II and 2009 harmonized criteria. Our results align with those of Shiferaw et al., who similarly found higher and lower prevalence rates of MetS based on the NCEP-ATP II and WHO criteria, respectively (24). We also found that metabolic syndrome was more prevalent in females (48%) compared to males (32%). Consistent with our findings, several studies have suggested that MetS is about twice as highly prevalent in females compared with males (26, 43). The higher prevalence of MetS in women may be due to their increased rates of obesity and body mass index. Additionally, women tend to have a higher proportion of upper body adiposity and abdominal obesity, including fat deposition in the buttock, hip, and leg regions, which are more common in women than in men.

There are several limitations in our meta-analysis. First, individual studies used different diagnostic criteria to measure metabolic syndrome, which contributed to heterogeneity and variability in the findings across studies. This led to disparities in the pooled prevalence between IDF, NCEP ATP III, 2009 harmonized, and WHO diagnostic criteria. Second, all the included studies used small sample sizes, which may slightly affect the robustness of the MetS prevalence estimates. Third, most of the studies included in this meta-analysis were single-center and facility-based, potentially limiting their generalizability to the broader population of Ethiopia. Lastly, our analysis was based on observational studies, and future cohort prospective studies may be needed to confirm our findings.

## Conclusions

This meta-analysis revealed that the prevalence of metabolic syndrome (MetS) among type 2 diabetic patients was 53%, with a higher prevalence observed in females compared to males (48% vs. 32%). Healthcare providers can play a crucial role in reducing the prevalence of MetS by implementing routine screening protocols, offering gender-specific interventions, and promoting awareness of lifestyle modifications. These strategies can effectively address the high prevalence of MetS among type 2 diabetic patients, ultimately improving patient outcomes and quality of life.

## Data Availability

The original contributions presented in the study are included in the article/supplementary material. Further inquiries can be directed to the corresponding author.

## References

[1] Executive summary of the third report of the national cholesterol education program (NCEP) expert panel on detection, evaluation, and treatment of high blood cholesterol in adults (Adult treatment panel III). Jama. (2001) 285:2486–97. doi: 10.1001/jama.285.19.2486 11368702

[2] Eckel RH Grundy SM Zimmet PZ . The metabolic syndrome. Lancet. (2005) 365:1415–28. doi: 10.1016/S0140-6736(05)66378-7 15836891

[3] Alberti KG Zimmet P Shaw J . Metabolic syndrome–a new world-wide definition. A Consensus Statement from the International Diabetes Federation. Diabetes Med. (2006) 23:469–80. doi: 10.1111/j.1464-5491.2006.01858.x 16681555

[4] Gami AS Witt BJ Howard DE Erwin PJ Gami LA Somers VK . Metabolic syndrome and risk of incident cardiovascular events and death: a systematic review and meta-analysis of longitudinal studies. J. Am. Coll. Cardiol. (2007) 49:403–14. doi: 10.1016/j.jacc.2006.09.032 17258085

[5] Alberti KG Zimmet P Shaw J . The metabolic syndrome–a new worldwide definition. Lancet. (2005) 366:1059–62. doi: 10.1016/S0140-6736(05)67402-8 16182882

[6] Cankurtaran M Halil M Yavuz BB Dagli N Oyan B Ariogul S . Prevalence and correlates of metabolic syndrome (MS) in older adults. Arch. Gerontol Geriatr. (2006) 42:35–45. doi: 10.1016/j.archger.2005.05.004 16046242

[7] Roden M Shulman GI . The integrative biology of type 2 diabetes. Nature. (2019) 576:51–60. doi: 10.1038/s41586-019-1797-8 31802013

[8] Chowdhury MZI Anik AM Farhana Z Bristi PD Mamun DMAA Uddin MJ . Prevalence of metabolic syndrome in Bangladesh: a systematic review and meta-analysis of the studies. BMC Public Health. (2018) 18:308. doi: 10.1186/s12889-018-5209-z 29499672 PMC5833131

[9] Hossain S Fatema K Ahmed KR Akter J Chowdhury HA Shahjahan M . Prevalence and determinants of metabolic syndrome among newly diagnosed type 2 diabetic subjects according to different criteria. Diabetes Metab. Syndr. (2015) 9:120–3. doi: 10.1016/j.dsx.2014.04.016 25470642

[10] Bowo-Ngandji A Kenmoe S Ebogo-Belobo JT Kenfack-Momo R Takuissu GR Kengne-Ndé C . Prevalence of the metabolic syndrome in African populations: A systematic review and meta-analysis. PloS One. (2023) 18:e0289155. doi: 10.1371/journal.pone.0289155 37498832 PMC10374159

[11] Zerga AA Bezabih AM . Metabolic syndrome and lifestyle factors among type 2 diabetes mellitus patients in Dessie Referral Hospital, Amhara region, Ethiopia. PloS One. (2020) 15:e0241432. doi: 10.1371/journal.pone.0241432 33137150 PMC7605694

[12] Bizuayehu Wube T Mohammed Nuru M Tesfaye Anbese A . A comparative prevalence of metabolic syndrome among type 2 diabetes mellitus patients in hawassa university comprehensive specialized hospital using four different diagnostic criteria. Diabetes Metab. Syndr. Obes. (2019) 12:1877–87. doi: 10.2147/DMSO.S221429 PMC6756827 31571962

[13] Gebremeskel GG Berhe KK Belay DS Kidanu BH Negash AI Gebreslasse KT . Magnitude of metabolic syndrome and its associated factors among patients with type 2 diabetes mellitus in Ayder Comprehensive Specialized Hospital, Tigray, Ethiopia: a cross sectional study. BMC Res. Notes. (2019) 12:1–7. doi: 10.1186/s13104-019-4609-1 31533851 PMC6751785

[14] Charkos TG Getnet M . Metabolic syndrome in patients with type 2 diabetes mellitus at Adama Hospital Medical College, Ethiopia: a hospital-based cross-sectional study. Front. Clin. Diabetes Healthc. (2023) 4:1165015. doi: 10.3389/fcdhc.2023.1165015 37396441 PMC10311433

[15] Gemeda D Abebe E Duguma A . Metabolic syndrome and its associated factors among type 2 diabetic patients in southwest Ethiopia, 2021/2022. J. Diabetes Res 2022. (2020) p:8162342. doi: 10.1155/2022/8162342 PMC9553723 36248224

[16] Kassie AM Abate BB Kassaw MW . Prevalence of overweight/obesity among the adult population in Ethiopia: a systematic review and meta-analysis. BMJ Open. (2020) 10:e039200. doi: 10.1136/bmjopen-2020-039200 PMC7412611 32764091

[17] Tadewos A AH Assegu D . Pattern of Metabolic Syndrome in relation to Gender among type-II DM patients in Hawassa University Comprehensive Specialized Hospital, Hawassa, Southern Ethiopia. Health Sci. J. (2017) 11:3. doi: 10.21767/1791-809X.1000509

[18] Shita A Teshome H Ayalew M Yesuf W Getachew D . Metabolic syndrome and its associated factors among type 2 diabetic patients in Mizan-Tepi University Teaching Hospital, Southwest Ethiopia Region. Front. Clin. Diabetes Healthcare. (2023) 4:1234674. doi: 10.3389/fcdhc.2023.1234674 PMC10542573 37790676

[19] Foroozanfar ZM Najafipour H Khanjani N Bahrampour A Ebrahimi H . The prevalence of metabolic syndrome according to different criteria and its associated factors in type 2 diabetic patients in kerman, Iran. Iran J. Med. Sci. (2015) 40:522–5. PMC4628143 26538781

[20] Nahar S RM Ullah M Debnath B Sultana N Farhad C . Prevalence of metabolic syndrome in newly diagnosed type 2 diabetes mellitus. Cardiovasc. J. (2011) 4:17.e25.

[21] Jacob B GA Jose R Antony T Sebastain S . Prevalence of metabolic syndrome in newly detected type 2 diabetes mellitus. Acad. Med. J. India. (2015) 3:8e12.

[22] Birarra MK Gelayee DA . Metabolic syndrome among type 2 diabetic patients in Ethiopia: a cross-sectional study. BMC Cardiovasc. Disord. (2018) 18:149. doi: 10.1186/s12872-018-0880-7 30016936 PMC6050670

[23] Saklayen MG . The global epidemic of the metabolic syndrome. Curr. Hypertens. Rep. (2018) 20:12. doi: 10.1007/s11906-018-0812-z 29480368 PMC5866840

[24] Shiferaw WS Akalu TY Gedefaw M Anthony D Kassie AM Misganaw Kebede W . Metabolic syndrome among type 2 diabetic patients in Sub-Saharan African countries: A systematic review and meta-analysis. Diabetes Metab. Syndr. (2020) 14:1403–11. doi: 10.1016/j.dsx.2020.07.013 32755843

[25] Biadgo B Melak T Ambachew S Baynes HW Limenih MA Jaleta KN . The Prevalence of Metabolic Syndrome and Its Components among Type 2 Diabetes Mellitus Patients at a Tertiary Hospital, Northwest Ethiopia. Ethiop J. Health Sci. (2018) 28:645–54. doi: 10.4314/ejhs.v28i5.16 PMC6308785 30607080

[26] James M Varghese TP Sharma R Chand S . Association between metabolic syndrome and diabetes mellitus according to international diabetic federation and national cholesterol education program adult treatment panel III criteria: a cross-sectional study. J. Diabetes Metab. Disord. (2020) 19:437–43. doi: 10.1007/s40200-020-00523-2 PMC7270215 32550195

[27] Demissie BM Girmaw F Amena N Ashagrie G . Prevalence of metabolic syndrome and associated factors among patient with type 2 diabetes mellitus in Ethiopia, 2023: asystematic review and meta analysis. BMC Public Health. (2024) 24:1128. doi: 10.1186/s12889-024-18580-0 38654186 PMC11040765

[28] Liberati A Altman DG Tetzlaff J Mulrow C Gotzsche PC Ioannidis JP . The PRISMA statement for reporting systematic reviews and meta-analyses of studies that evaluate health care interventions: explanation and elaboration. J. Clin. Epidemiol. (2009) 62(10):e1-34. doi: 10.1016/j.jclinepi.2009.06.006 19631507

[29] Alberti KG Eckel RH Grundy SM Zimmet PZ Cleeman JI Donato KA . Harmonizing the metabolic syndrome: a joint interim statement of the International Diabetes Federation Task Force on Epidemiology and Prevention; National Heart, Lung, and Blood Institute; American Heart Association; World Heart Federation; International Atherosclerosis Society; and International Association for the Study of Obesity. Circulation. (2009) 120:1640–5. doi: 10.1161/CIRCULATIONAHA.109.192644 19805654

[30] Alberti KG Zimmet PZ . Definition, diagnosis and classification of diabetes mellitus and its complications. Part 1: diagnosis and classification of diabetes mellitus provisional report of a WHO consultation. Diabetes Med. (1998) 15:539–53. doi: 10.1002/(SICI)1096-9136(199807)15:7<539::AID-DIA668>3.0.CO;2-S 9686693

[31] Wells GA . The Newcastle-Ottawa Scale (NOS) for assessing the quality of nonrandomized studies in meta-analyses. 3rd Symposium on Systematic Reviews: Beyond the Basics. Oxford, UK (2000). Available online at: https://cmr.cochrane.org/?CRGReportID=2972.

[32] DerSimonian R Laird N . Meta-analysis in clinical trials. Control Clin. Trials. (1986) 7:177–88. doi: 10.1016/0197-2456(86)90046-2 3802833

[33] Barendregt JJ Doi SA Lee YY Norman RE Vos T . Meta-analysis of prevalence. J. Epidemiol. Community Health. (2013) 67:974–8. doi: 10.1136/bmj.327.7414.557 23963506

[34] Cochran WG . The combination of estimates from different experiments. Biometrics. (1954) 3:101–29. doi: 10.2307/3001666

[35] Higgins JP Thompson SG Deeks JJ Altman DG . Measuring inconsistency in meta-analyses. BMJ. (2003) 327:557–60. doi: 10.1136/bmj.327.7414.557 PMC192859 12958120

[36] Egger MD Davey Smith G Schneider M Minder C . Bias in meta-analysis detected by a simple, graphical test. BMJ. (1997) 315:629–34. doi: 10.1136/bmj.315.7109.629 PMC2127453 9310563

[37] Woyesa SB Hirigo AT Wube TB . Hyperuricemia and metabolic syndrome in type 2 diabetes mellitus patients at Hawassa university comprehensive specialized hospital, South West Ethiopia. BMC Endocrine Disord. (2017) 17:76. doi: 10.1186/s12902-017-0226-y PMC5728062 29233152

[38] Lamat H Sauvant-Rochat MP Tauveron I Bagheri R Ugbolue UC Maqdasi S . Metabolic syndrome and pesticides: A systematic review and meta-analysis. Environ. pollut. (2022) 305:119288. doi: 10.1016/j.envpol.2022.119288 35439599

[39] Ambachew S Endalamaw A Worede A Tegegne Y Melku M Biadgo B . The prevalence of metabolic syndrome in Ethiopian population: A systematic review and meta-analysis. J. Obes 2020. (2020) p:2701309. doi: 10.1155/2020/2701309 PMC7803160 33489358

[40] Ebtekar F Dalvand S Gheshlagh RG . The prevalence of metabolic syndrome in postmenopausal women: A systematic review and meta-analysis in Iran. Diabetes Metab. Syndr. (2018) 12:955–60. doi: 10.1016/j.dsx.2018.06.002 29891195

[41] Kwasny C Manuwald U Kugler J Rothe U . Systematic review of the epidemiology and natural history of the metabolic vascular syndrome and its coincidence with type 2 diabetes mellitus and cardiovascular diseases in different european countries. Horm. Metab. Res. (2018) 50:201–8. doi: 10.1055/s-0043-122395 29183091

[42] Mogre V Salifu ZS Abedandi R . Prevalence, components and associated demographic and lifestyle factors of the metabolic syndrome in type 2 diabetes mellitus. J. Diabetes Metab. Disord. (2014) 13:80. doi: 10.1186/2251-6581-13-80 25054102 PMC4106220

[43] Khanal P Patil BM Mandar BK Duyu T . Network pharmacology-based assessment to elucidate the molecular mechanism of anti-diabetic action of Tinospora cordifolia. Clin. Phytoscience. (2019) 5:35. doi: 10.1186/s40816-019-0131-1

